# A Discussion of the Contemporary Prediction Models for Atrial Fibrillation

**DOI:** 10.18103/mra.v11i10.4481

**Published:** 2023-10-25

**Authors:** Michael A. Rosenberg, Joseph Adewumi, Ryan G. Aleong

**Affiliations:** 1 Department of Cardiac Electrophysiology, University of Colorado, Aurora, Colorado, USA

**Keywords:** Atrial Fibrillation, Clinical Risk Scores, Genome Wide Association Studies, Polygenic Risk Score

## Abstract

Atrial Fibrillation is a complex disease state with many environmental and genetic risk factors. While there are environmental factors that have been shown to increase an individual’s risk of atrial fibrillation, it has become clear that atrial fibrillation has a genetic component that influences why some patients are at a higher risk of developing atrial fibrillation compared to others. This review will first discuss the clinical diagnosis of atrial fibrillation and the corresponding rhythm atrial flutter. We will then discuss how a patients’ risk of stroke can be assessed by using other clinical co-morbidities. We will then review the clinical risk factors that can be used to help predict an individual patient’s risk of atrial fibrillation. Many of the clinical risk factors have been used to create several different risk scoring methods that will be reviewed. We will then discuss how genetics can be used to identify individuals who are at higher risk for developing atrial fibrillation. We will discuss genome-wide association studies and other sequencing high-throughput sequencing studies. Finally, we will touch on how genetic variants derived from a genome-wide association studies can be used to calculate an individual’s polygenic risk score for atrial fibrillation. An atrial fibrillation polygenic risk score can be used to identify patients at higher risk of developing atrial fibrillation and may allow for a reduction in some of the complications associated with atrial fibrillation such as cerebrovascular accidents and the development of heart failure. Finally, there is a brief discussion of how artificial intelligence models can be used to predict which patients will develop atrial fibrillation.

## Introduction

The goal of this manuscript is to discuss atrial fibrillation. The diagnosis of atrial fibrillation and related heart rhythm, atrial flutter, will be discussed. The risk factors for cerebrovascular accidents will be discussed. The manuscript will then discuss clinical and genetic risk scores that can predict which patients will develop atrial fibrillation. Finally, the manuscript will briefly discuss artificial intelligence tools that can be used to assess which patients will develop atrial fibrillation.

### Clinical Diagnosis of Atrial Fibrillation

Atrial fibrillation is the most common cardiac arrhythmia and can be associated with significant cardiac morbidity and mortality. Cerebrovascular accidents and transient ischemic attacks have been associated with atrial fibrillation and anticoagulation is recommended for many patients depending on their co-morbidities. It is important to differentiate between valvular and non-valvular atrial fibrillation to determine the appropriate type of anticoagulation. Valvular atrial fibrillation occurs in the setting of moderate to severe mitral stenosis or in patients who have artificial or mechanical heart valves. Non-valvular atrial fibrillation does not imply that patients do not have valvular heart disease. For patients with valvular atrial fibrillation, anticoagulation with warfarin is recommended. For patients with non-valvular atrial fibrillation, either warfarin or non-vitamin K oral anticoagulants (NOACs) can be used for cerebrovascular accident and transient ischemic attack prevention. The correct type of anticoagulation for patients with non-valvular atrial fibrillation can be determined by using the CHA_2_DS_2_-VASc score (Table 1) with oral anticoagulation recommended for men with a score equal to or greater than 2 or 3 in women. Anticoagulation for patients with atrial fibrillation is more fully discussed in the Guidelines for the Management of Patients with Atrial Fibrillation with the most recent guidelines written in 2019.^1^

**Figure 1:**
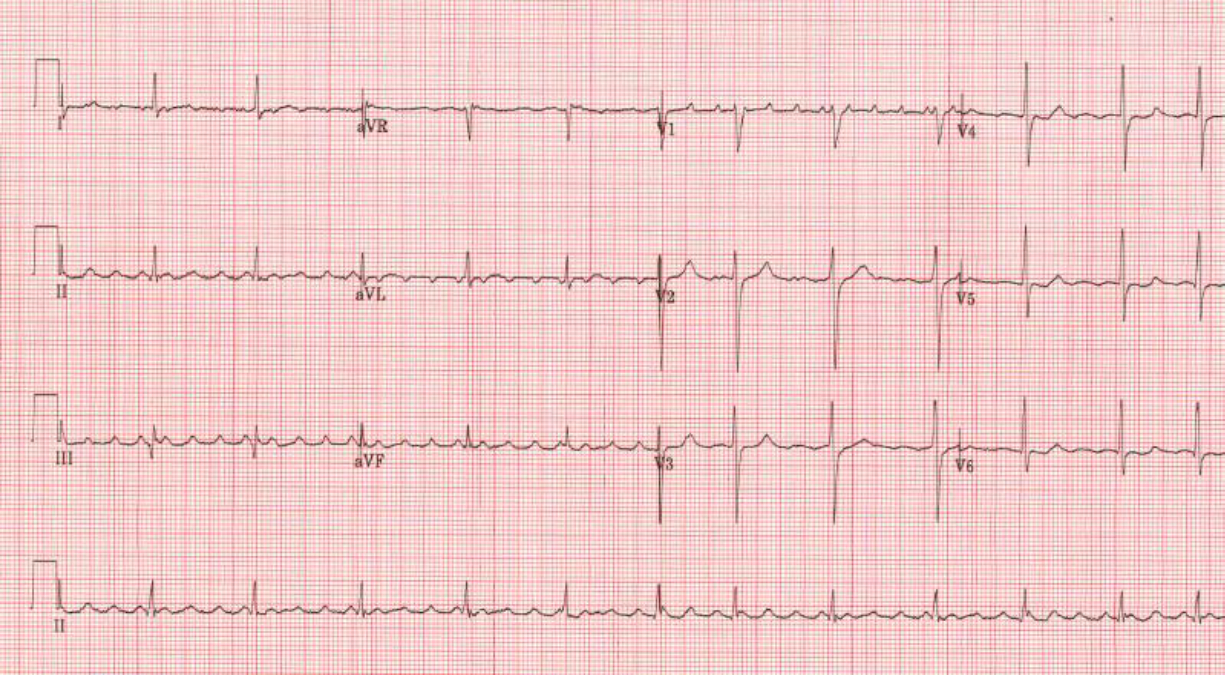


**Figure 2:**
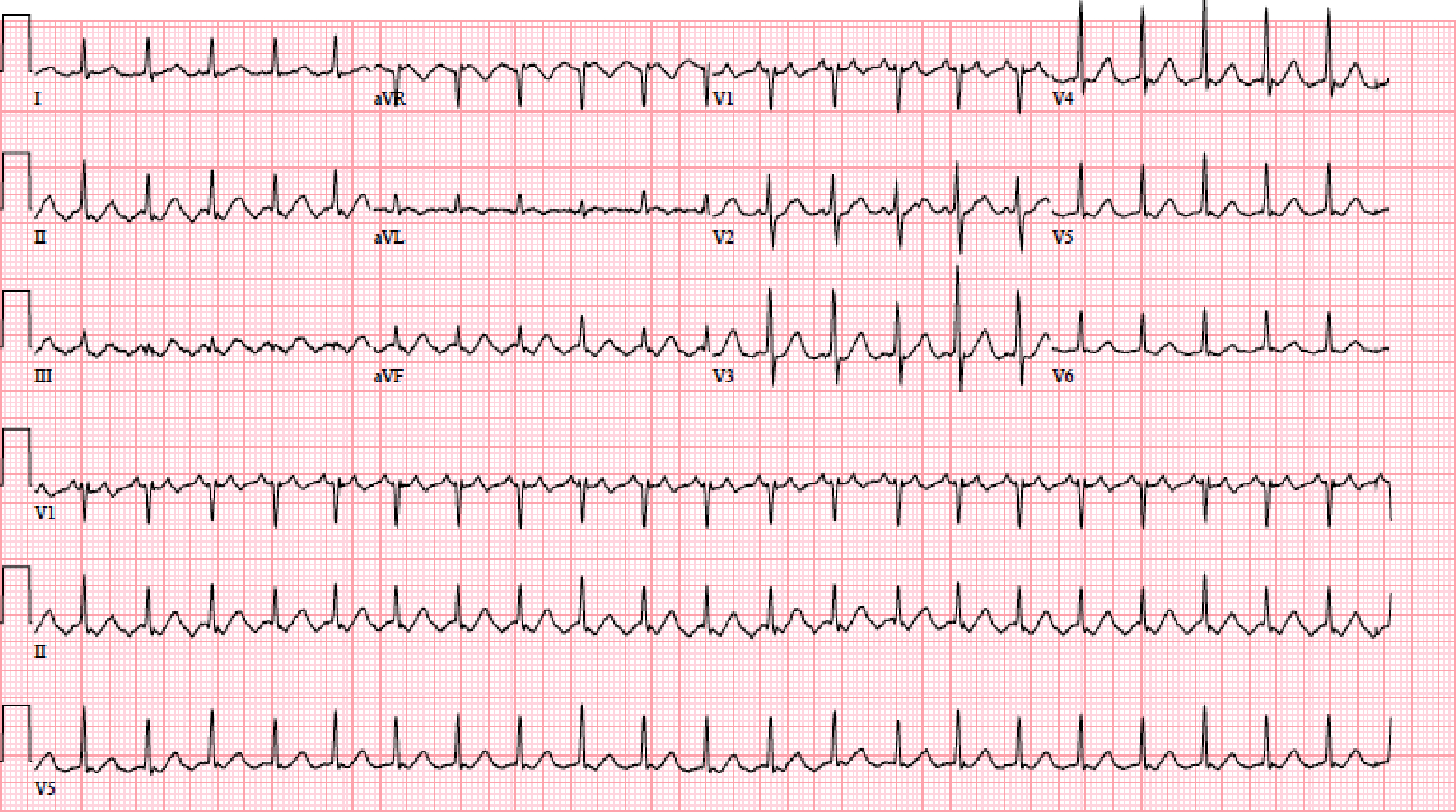


Atrial fibrillation is diagnosed using an electrocardiogram (ECG) or cardiac monitor that shows no organized atrial activity and typically an irregularly, irregular ventricular rhythm (Figure 1). A related heart rhythm is atrial flutter. On electrocardiogram, atrial flutter is characterized by a saw tooth pattern in the inferior ECG leads and depending on whether it arises from left or right atrium, the atrial flutter will be either positive across the precordial ECG leads (left atrium) or shift from positive to negative or negative to positive in the precordial leads (right atrium). Figure 2 shows atrial flutter arising from the right atrium with positive flutter waves in V1 shifting to negative flutter waves in V6.

### Clinical Risk Scores for Atrial Fibrillation

There have been several risk scores developed that use clinical characteristics to predict which patients will develop atrial fibrillation. One of the first described clinical risk scores was derived from the Framingham Heart Study which assessed 4764 individuals from 1968 to 1987 to investigate who would develop atrial fibrillation over ten years of follow up.^2^ A risk score was developed that uses age, the PR interval on the electrocardiogram, body-mass index, systolic blood pressure, hypertension and the presence of heart failure or a murmur to predict an individual’s likelihood of developing atrial fibrillation. Men and women with a higher risk score had a 27% risk of developing atrial fibrillation over ten years of follow up. Another published risk scoring system is the CHARGE-AF risk score that includes age, height, weight, blood pressure, smoker status, diabetes, heart failure and the use of antihypertensive medications.^3^ The CHARGE-AF or Cohorts for Heart and Aging Research in Genomic Epidemiology AF consortium was a pooled database comprised of 18, 556 patients from the Atherosclerosis Risk in Communities, the Cardiovascular Health Study and Framingham Heart Study. The Cohorts for Heart and Aging Research in Genomic Epidemiology AF risk score is calculated by using the following equation: 0.508 × age (5 years) + 0.248 × height (10 cm) + 0.115 × weight (15kg) + 0.197 × systolic blood pressure (20 mm Hg) − 0.101 × diastolic blood pressure (10 mm Hg) + 0.359 × current smoker + 0.349 × antihypertensive medication + 0.237 × diabetes + 0.701 × congestive heart failure + 0.496 × myocardial infarction.^3,4^

Finally, the last important clinical risk score that may be useful clinically is the Taiwan Risk Score that was developed using the medical records of 7,220,654 individuals.^5^ The risk score is outlined in Table 2. The risk of incident atrial fibrillation increased from 0.05%/year with a score of −2 to 6.95%/year for those with a score >14. While the Taiwan score was developed in an Asian population, this score has many of the same clinical characteristics of other risk scores and can be used for many patients. Like many of the other risk scores, the Taiwan Risk Score uses age as a major predictor of incidence atrial fibrillation.

### Artificial Intelligence Models to Predict Atrial Fibrillation

Risk scores provide an interpretable approach to predict atrial fibrillation in such a way that clinicians can understand how risk factors can be combined to predict risk. However, with the advances in artificial intelligence, investigators have proposed more complex models to predict incident atrial fibrillation. For example, Tiwari et al., applied machine-learning models to electronic health record data across over 2.2 million subjects, using the 200 most common diagnoses, medications, and procedures, and demonstrated superiority compared with a model with only known risk factors^6^. Ambale-Vankatesh et al., used a random forest model applied to clinical data from the Multi-Ethnic Study of Atherosclerosis clinical cohort to predict incident atrial fibrillation, noting that age, creatinine, and ankle-brachial index were the strongest predictors^7^. Several contemporary studies have used 12-lead ECG in sinus rhythm to predict future atrial fibrillation. Raghunath et al., applied deep learning to 12-lead electrocardiogram data to predict incident atrial fibrillation, as well as atrial fibrillation-related stroke^8^. Attia et al., also developed deep-learning models applied to sinus rhythm electrocardiogram to predict future atrial fibrillation^9^, and demonstrated feasibility in guiding screening for atrial fibrillation in a prospective analysis^10^. At balance in these various approaches has been the improvement in predictive accuracy with use of artificial intelligence methods, at the cost of a loss in interpretability that comes with use of black-box models^11^, a trade-off that was directly demonstrated by Simon et al., in a study of drug-induced QT prolongation^12^. The issue of understanding what data is actually being used to make predictions, and how it relates to known physiological and pathophysiological properties of atrial fibrillation is also relevant in genetic prediction models.

### Atrial Fibrillation and Genetics

There is now a large recognition that genetics plays a significant role in atrial fibrillation. There are multiple studies showing that atrial fibrillation can be familial.^13,14^ The Framingham Heart Study showed that if atrial fibrillation is present in family members, there is a 40% increased risk of developing atrial fibrillation.^15^ Therefore, atrial fibrillation has become recognized as a cardiac arrhythmia with a large genetic component.

### Genetic Risk Scores for Atrial Fibrillation

Genetic loci that are linked to atrial fibrillation have been uncovered using genome-wide association studies. These genome-wide association studies have revealed that there are complex biological processes that are associated with atrial fibrillation. The initial genome-wide association studies published in 2007 demonstrated a genetic variant at the chromosome 4q25 locus near the *PITX2* gene, which is a transcription factor that drives early tissue development and left-right cardiac patterning. The locus near to *PITX2* demonstrated a 40–60% increased risk of developing atrial fibrillation in affected individuals.^16^ PITX2 seems to alter potassium and calcium channels that shorten the action potential and may make patients more susceptible to having atrial fibrillation. Interestingly, there are other genes that are associated with atrial fibrillation that may interact with PITX2. These genes include TBX5, HCN4 and CAV-1.^17,18^ Further genome-wide association studies have found loci associated with ion channels, electrical signaling and structural aspects of the cardiomyocyte. A recent genome-wide association studies found 149 genetic loci associated with atrial fibrillation in 150,272 European and Japanese individuals identified 35 new susceptibility loci.^19^ Interestingly, the *IL6R* gene was identified as a possible causal gene, which may explain the association between inflammation and atrial fibrillation. The authors also found that the transcription factor, estrogen-related receptor gamma, had enriched binding among the genetic loci to increase the expression of genes that are associated with atrial fibrillation development. The authors then identified a polygenic risk score derived from subjects with multiple ancestries (Japanese and European) that was associated with an earlier age of onset of atrial fibrillation and was associated with several stroke phenotypes, which may reveal that the AF-Polygenic Risk Score may be associated with clinically silent atrial fibrillation.

### Clinical and Genetic Risk Scores

Given that atrial fibrillation is a complex medical condition with clinical and genetic risk factors, there have been studies using both risk factor schemes. Marston et al. published their work analyzing 36,662 individuals from four of the Thrombolysis in Myocardial Infarction trials to investigate the risk of atrial fibrillation.^20^ They used the CHARGE-AF risk score and a Polygenic Risk Score for atrial fibrillation to look at the incidence of atrial fibrillation in patients with no prior atrial fibrillation. The Polygenic Risk Score for atrial fibrillation augmented the risk of atrial fibrillation regardless of the Cohorts for Heart and Aging Research in Genomic Epidemiology AF score. For example, in patients with a high Cohorts for Heart and Aging Research in Genomic Epidemiology AF score, the risk of developing atrial fibrillation increased from 3.3% to 8.7% over three years of follow up. Even in patients with a low clinical risk of atrial fibrillation, the risk of atrial fibrillation increased from 1.3% to 3.3%. Another means of using a PRS for atrial fibrillation is to measure the Polygenic Risk Score in patients undergoing ablation for atrial fibrillation. Al-Kaisey et al. showed that a higher Polygenic Risk Score for atrial fibrillation in patients undergoing ablation for atrial fibrillation was associated with more atrial arrhythmias at follow up over one to two years.^21^ Furthermore, a higher Polygenic Risk Score for atrial fibrillation was associated with a greater degree of atrial structural and electrical remodeling. Based on these two trials, it does appear that a Polygenic Risk Score for atrial fibrillation can improve care of patients who have atrial fibrillation.

### Potential Insights into Genetic Risk of Atrial Fibrillation

Despite proposed mechanisms for certain genetic predictors of atrial fibrillation, the underlying mechanism of genetic-mediated risk remains somewhat elusive. One interesting potential link lies in a somewhat unrecognized clinical risk factor for atrial fibrillation, which is body size or height. A number of observational studies have demonstrated the association of increased body size with risk of atrial fibrillation^22–24^, including a study by Rosenberg et al., which demonstrated that the increased risk of atrial fibrillation among men was entirely explained by the increased height in men compared with women^25^. Interestingly, in one of the largest genome wide association studies to date on atrial fibrillation, the trait with the greatest amount of pleiotropy among associated variants with atrial fibrillation was height^26^, and Mendelian randomization studies have also indicated that genetic height was a significant predictor of atrial fibrillation^27–29^. Uncovering mechanistic explanations remains an open question for these studies, and while height, genetically determined or otherwise, is an unmodifiable risk factor, it raises potential for applications to improvement of clinical prediction of atrial fibrillation.

## Conclusion

The diagnosis and management of atrial fibrillation has continued to evolve over the past ten years. There are many clinical risk scores that are easy to estimate a patient’s risk of developing atrial fibrillation. The Cohorts for Heart and Aging Research in Genomic Epidemiology AF risk score has been validated in many other publications, but other scores such as the Taiwan Risk Score are easy to use when seeing patients in any hospital or clinic setting. Polygenic risk scores can better identify which patients are at greatest risk for developing atrial fibrillation and how treatment strategies, such as ablation, can decrease the duration and frequency of atrial fibrillation. Finally, diagnosis and treatment of atrial fibrillation is rapidly changing and may improve the care of these patients.

## Figures and Tables

**Table 1:** 

Clinical characteristic	Score
Congestive Heart Failure	1
Hypertension	1
Age, 65 to 74 years	1
Age, greater than 75 years	2
Diabetes mellitus	1
Prior cerebrovascular accident, transient ischemic attack or thromboembolism	2
Vascular disease	1
Female	1

**Table 2:** 

Age (years)	Score
40-44	−2
45-49	−1
50-54	0
55-59	1
60-64	2
65-69	3
70-74	4
75-79	5
>80	80
Male sex	1
Hypertension	1
Coronary artery disease	1
ESRD	1
Alcoholism	1
Total Score	−2 to 15
